# The nitrogen-dependent GABA pathway of tomato provides resistance to a globally invasive fruit fly

**DOI:** 10.3389/fpls.2023.1252455

**Published:** 2023-12-07

**Authors:** Hao Li, Yuan Zhang, Hu Li, Gadi V. P. Reddy, Zhihong Li, Fajun Chen, Yucheng Sun, Zihua Zhao

**Affiliations:** ^1^ Department of Plant Biosecurity & Ministry of Agriculture and Rural Affairs (MARA) Key Laboratory of Surveillance and Management for Plant Quarantine Pests, College of Plant Protection, China Agricultural University, Beijing, China; ^2^ Sanya Institute of China Agricultural University, Sanya, China; ^3^ Department of Entomology, Louisiana State University, Baton Rouge, LA, United States; ^4^ Department of Entomology, College of Plant Protection, Nanjing Agricultural University, Nanjing, China; ^5^ National Key Lab Integrated Management Pest Insects, Institute of Zoology, Chinese Academy Science, Beijing, China

**Keywords:** nitrogen, γ-aminobutyric acid, *Bactrocera dorsalis*, interaction, primary metabolism, plant defense

## Abstract

**Introduction:**

The primary metabolism of plants, which is mediated by nitrogen, is closely related to the defense response to insect herbivores.

**Methods:**

An experimental system was established to examine how nitrogen mediated tomato resistance to an insect herbivore, the oriental fruit fly (*Bactrocera dorsalis*). All tomatoes were randomly assigned to the suitable nitrogen (control, CK) treatment, nitrogen excess (NE) treatment and nitrogen deficiency (ND) treatment.

**Results:**

We found that nitrogen excess significantly increased the aboveground biomass of tomato and increased the pupal biomass of *B. dorsalis*. Metabolome analysis showed that nitrogen excess promoted the biosynthesis of amino acids in healthy fruits, including γ-aminobutyric acid (GABA), arginine and asparagine. GABA was not a differential metabolite induced by injury by *B. dorsalis* under nitrogen excess, but it was significantly induced in infested fruits at appropriate nitrogen levels. GABA supplementation not only increased the aboveground biomass of plants but also improved the defensive response of tomato.

**Discussion:**

The biosynthesis of GABA in tomato is a resistance response to feeding by *B. dorsalis* in appropriate nitrogen, whereas nitrogen excess facilitates the pupal weight of *B. dorsalis* by inhibiting synthesis of the GABA pathway. This study concluded that excess nitrogen inhibits tomato defenses in plant-insect interactions by inhibiting GABA synthesis, answering some unresolved questions about the nitrogen-dependent GABA resistance pathway to herbivores.

## 1 Introduction

As an important inorganic nutrient of plants, nitrogen participates in various physiological metabolic processes, being a main component of proteins, nucleic acids and secondary metabolites. The nitrogen concentration in plants is influenced by the environmental nitrogen level. Generally, low nitrogen will reduce the concentrations of amino acids and other nitrogen-containing compounds (e.g., chlorophyll) in plants. These changes will affect the carbon and nitrogen assimilation of plants and limit the accumulation of plant biomass (Schlüter et al., 2012). Plants grown with limited resources may prefer to produce more defensive compounds (Wilkens et al., 1996). High nitrogen significantly promotes the growth of plants with adequate environmental resources, but plants in high-nitrogen environments have rich cytoplasm and thinner cuticles (Jauset et al., 2000). High nitrogen fertilization leads to a reduction in the content of lignin, which decreases plant defenses by mediating the thickness of the plant’s physical barrier (Zhang et al., 2017). Nitrogen fertilizers may not directly affect the physiology of insects. High nitrogen would shift plant physiology (e.g., amino acid biosynthesis and tricarboxylic acid cycle) to affect the nutritional conditions for herbivorous insects (Foyer et al., 2011). Herbivores prefer high-nitrogen plants, and the population size of herbivores increases significantly in high-nitrogen plants (Li et al., 2016). The population of *Bemisia tabaci* increases with increasing amounts of nitrogen fertilizer under field conditions (Bi et al., 2001). High nitrogen also increases the egg survival rate and pupal exuviae size of *Trialeurodes vaporariorum* on tomato plants (Jauset et al., 2000).

A high-nitrogen environment supplies plants with more of this macronutrient, and insects can obtain more suitable nutrients under this nutrient condition. The carbon-nutrient balance hypothesis (Bryant et al., 1983) holds that high nitrogen affects the energy distribution of primary or secondary metabolism in plants. High-nitrogen environments induce plants to invest more resources in growth and inhibit the synthesis of secondary metabolites (Zheng et al., 2021). The expression of the plant defense response induced by herbivores may also be affected by N availability (Ren et al., 2013).

Ammonium and nitrate are the main forms of inorganic nitrogen that plants absorb. Nitrogen availability can change the basic biochemistry and other physiological activities of plants by regulating the primary metabolism of nitrogen and carbon (Wang et al., 2019). Ammonium is usually transformed into amino acids in plants, a process is called nitrogen assimilation(Ghanem et al., 2011). Amino acids, which are the first stable products of nitrogen assimilation, are significantly affected by the nitrogen regime and nitrogen source (Kant et al., 2011). As an important product of nitrogen assimilation, glutamic acid is a very active amino acid, being the starting point for the synthesis of other amino acids and can be rapidly converted into other nitrogenous compounds in plant cells. Glutamic acid metabolism plays a central role in plant nitrogen metabolism, including the biosynthesis of amino acids with key roles in plant defense, such as γ-aminobutyric acid (GABA) (Kan et al., 2015). Gamma-aminobutyric acid, a four-carbon nonprotein amino acid, exists in many organisms. In plants, GABA is considered a new plant growth regulator (Li et al., 2023). GABA can promote growth and improve plant resistance to stress by participating in signal transmission, regulating the stability of the plant antioxidant system, promoting photosynthesis and maintaining carbon and nitrogen metabolism(Shelp et al., 1999, Li and Zhou, 2022).

The entanglement between primary metabolism regulation and defense responses is a puzzling theme in plant science. Although many studies have focused on the effects of nitrogen on plant metabolism and the biological indicators of insect populations, the mechanism of metabolic changes in plant-insect interactions mediated by high nitrogen remains unclear. We hypothesized that high nitrogen can promote insect herbivore by changing the primary metabolism of plants. Tomato is an important crop and an excellent model plant for plant physiology research (Thaler et al., 2012). The oriental fruit fly, *Bactrocera dorsalis* (Hendel) (Diptera: Tephritidae), is a famous invasive agricultural pest that has caused serious losses in the global agricultural economy (Vargas et al., 2015). The main goal of this study is to address how high nitrogen affects the interaction between tomato and *B. dorsalis* by using metabolomics technology.

## 2 Materials and methods

### 2.1 The experimental models

Tomato plants (*Solanum lycopersicum* L. cultivar ‘Xia Luote’) were used in the experiments. Tomato plants that were 22 days old were transplanted from seedling plates into 25-cm-diameter pots (one plant per pot) containing a mixture of soil and vermiculite (7:3, v:v). From May through August, the greenhouse had a natural light-dark cycle with a constant temperature of 28°C. All tomatoes were randomly assigned to the suitable nitrogen (control, CK) treatment, nitrogen excess (NE) treatment and nitrogen deficiency (ND) treatment. The NE treatment was 500 mg/L CO(NH_2_)_2_ fertilizer. The CK treatment was a suitable nitrogen level of 250 mg/L CO(NH_2_)_2_ (Stout et al., 1998; Ding et al., 2020). The ND treatment was 0 mg/L CO(NH_2_)_2_. The same concentrations of potassium and phosphorus were added to all groups to replenish the potassium and phosphorus. Fertilizer (400 mL) was added four times once a week when the plants set fruit (12th week of growth), the stage at which leaf nitrogen content drops rapidly. These tomato plants and fruits were used at the red stage (6 days after breaker). The biomass of tomato shoots and roots was recorded after oven-drying the sample to a constant weight at 80°C (after 105°C for 20 min).

### 2.2 *B. dorsalis* infestation experiment

The *B. dorsalis* individuals were developed from larvae that were collected in rotten fruit in Guangdong Province. Before the experiment, the *B. dorsalis* population was raised in an intelligent artificial climate chamber (model: RXZ-500B) for 60 successive generations to lessen the potential impacts of environmental influence from the locality. The conditions of the climate chamber were 28°C ± 1°C, 70 ± 5% RH and 14 L:10 D cycle. Eggs of *B. dorsalis* were obtained utilizing an artificial egg extractor coated with mango juice. Larvae were fed an artificial diet consisting of 125 g sucrose, 31 g beer yeast, 235 g wheat bran, 2.1 g antibiotic, and 600 mL H_2_O. It took 8-10 days from egg hatching to larval maturity. The mature larvae sprang to the damp, 2-5-cm-deep sand (60%-70% water content) to pupate. The adult flies emerged after 7-8 days and were cultured in insect rearing cages (25 cm×25 cm×50 cm) using an artificial diet consisting of 75% sucrose and 25% peptone.

A simulated *B. dorsalis* infection experiment was carried out in a greenhouse. Six groups of treatments were set up to simulate *B. dorsalis* infection under laboratory conditions. CK group: healthy fruits without infection in suitable nitrogen (CK) treatment. CKPI group: fruits with infection in suitable nitrogen treatment. NE group: healthy fruits without infection in nitrogen excess (NE) treatment. NEPI group: fruits with infection in nitrogen excess treatment. ND group: healthy fruits without infection in nitrogen deficiency (ND) treatment. NDPI group: fruits with infection in nitrogen deficiency (ND) treatment. Five tomato plants with red fruit were selected in each group, and each plant was covered with nylon mesh (50 cm×50 cm×100 cm) to form an independent, small ecological environment. Pest injury treatment: Fifty sexually mature adults with 25 of each sex were released into each nylon mesh. Each fruit was checked to determine whether it was laid eggs by the *B. dorsalis* evert for six hours. Fruit samples were taken at 48 hours after spawning on fruits. All samples were immediately frozen in liquid nitrogen and stored at -80°C until needed for analysis.

### 2.3 Metabolomic analysis

#### 2.3.1 Sample extraction

A mixture comprising methanol, acetonitrile, and water (v:v:v = 2:2:1, LC–MS grade, Marda) was used as an extract solvent. The extracted ratio was 0.05 g of sample to 1 ml of solvent. The extracted solution was vortexed for 30 s and sonicated for 30 min at low temperature. After standing at -20°C for 10 min, the solution was centrifuged for 20 min at 14000 g at 4°C. The supernatant was transferred into an LC-MS vial for detection. Equivalent amounts of supernatants from all samples were mixed as QC (quality control) samples for testing.

#### 2.3.2 LC-MS analysis

Qualitative and quantitative analyses were performed using an Agilent 1290 Infinity LC ultrahigh-pressure liquid chromatograph (Agilent, USA) and an AB Triple TOF 6600 mass spectrometer (AB SCIEX, USA) with XCMS and SIMCA-P. Liquid chromatography was performed using a HILIC column (1.7 μm, 2.1 mm ×100 mm, Waters, USA). Detailed LC-MS analytical methods are provided in the **Supplementary Material.**

#### 2.3.3 Data preprocessing

The raw data in Wiff format were converted into mzXML format by ProteoWizard software, after which XCMS was used to perform retention time correction, peak identification, peak extraction, peak integration, and peak alignment. SIMCA software was used for multivariate statistical analyses and differential metabolite screening. HMDB and KEGG were used for metabolite identification.

### 2.4 Feeding validation experiment

Larvae were fed fruits of different treatments: positive control group: CK fruits with infested treatment; negative control group: CK fruits with healthy treatment; and two treatment groups: nitrogen-excess fruits with healthy treatment and nitrogen-excess fruits with infestation. In the healthy treatment, tomatoes received no treatment. In infested treatments, fruits were collected at 48 hours after oviposition on fruits by females. After different treatments, fruits were inoculated with 0.1 ml of solution with approximately 150 *B. dorsalis* eggs. Inoculation was performed by making a 2-mm-deep, 5-mm-long incision in the surface of fruit using a sterile surgical blade. The egg solution was transferred inside the incision using a sterile 1 ml injector. We observed and recorded the pupal weight of *B. dorsalis* on the fifth day after pupation.

### 2.5 GABA function verification experiment

After tomato plants that were 22 days old were transplanted from seedling plates into pots, 60 plants were divided into two groups. In the CK group, plants were sprayed with water once a week. In the GABA group, plants were sprayed with 5 mmol/L GABA solution. The biomass of plants was recorded after 14 days of cultivation under the same environmental conditions. *B. dorsalis* larvae were fed different artificial diets containing GABA. The two concentrations of the experimental diets were 0.5 µmol/g and 0.7 µmol/g GABA. The CK group was fed general artificial diets. The pupal weight was recorded on the fifth day after pupation.

### 2.6 Statistical analysis

One-way factorial ANOVA was used to analyze the differences in biomass of tomato or *B. dorsalis*. Levene’s test was used for variance homogeneity. Tukey’s HSD or Dunnett’s T3 test was used for *post hoc* analysis to detect significant differences at P < 0.05. All analyses were performed using SPSS software 13.0 (SPSS Inc., Chicago, IL, United States). The Kyoto Encyclopedia of Genes and Genomes (KEGG) database (http://www.kegg.jp/ (Release 97.0, January 1, 2021)) was used to do metabolic pathway enrichment using metabolites mapped to the database. Origin 2021 software (OriginLab Inc., Northampton, United States) was used for cluster analysis and Venn analysis.

## 3 Results

### 3.1 Effects of nitrogen on tomato and *B. dorsalis* biomass accumulation and fruit metabolism

**Figure 1:**
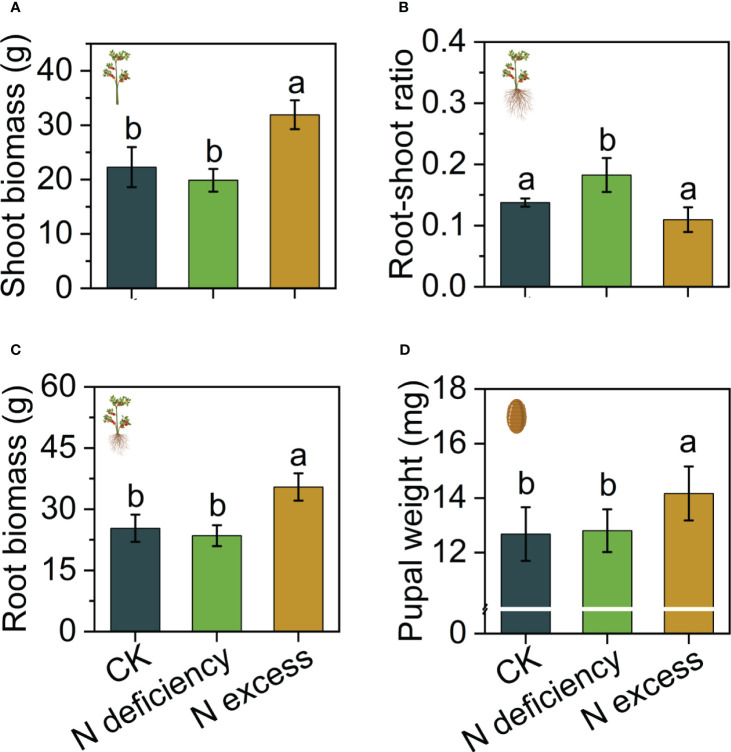
Effects of nitrogen on tomato and (*B*) *dorsalis* biomass accumulation. **(A)** The biomass of per tomato shoot. **(B)** The root/shoot ratio of per tomato. **(C)** The total biomass of per tomato. **(D)** The pupal weight of (*B*) *dorsalis* in infested fruits. The different letters in figure indicate significant differences between different treatments (P < 0.05).

Nitrogen excess significantly increased shoot biomass (F_2,6 _= 9.759, P < 0.05) and total biomass of tomato (F_2,6 _= 8.597, P < 0.05) (**Figures 1A, C**), while the root/shoot ratio showed ` nitrogen deficiency has the higher ratio than CK and nitrogen excess group(F = 6.66, P < 0.05) (**Figure 1B**). Additionally, giving excess nitrogen to the tomato increased the weight of pupae by 11% compared with theCK and nitrogen deficiency group (F_2,326 _= 91.517, P < 0.01) (**Figure 1D**).

**Figure 2:**
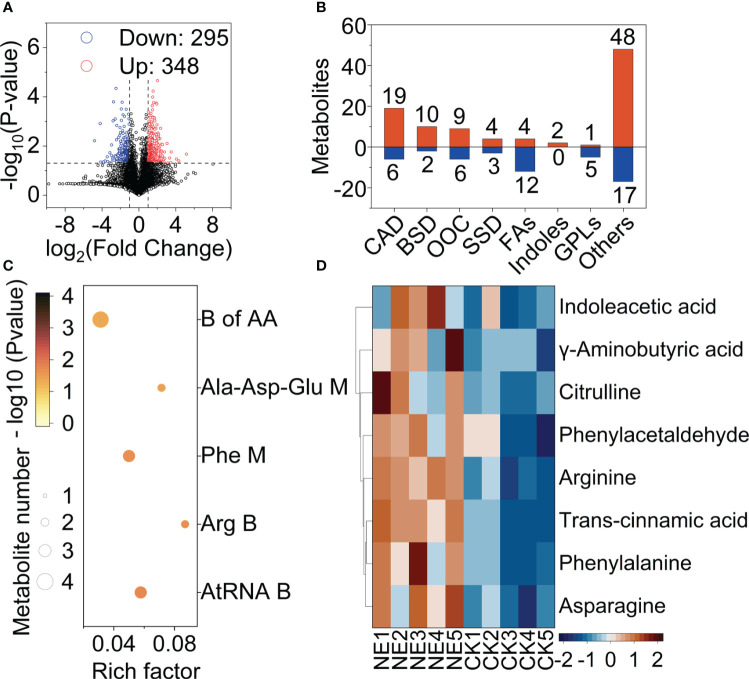
Overview of fruit metabolism changes under nitrogen excess (NE) and CK tomatoes. **(A)** Volcano map: the horizontal coordinate indicates the change in the metabolites (log_2_ fold change), and the vertical coordinate indicates the significance level (-log_10_ (P value)). The upregulated metabolites and downregulated metabolites are presented by red and blue dots, respectively. **(B)** Grouping classification of the upregulated and downregulated differential metabolites. CAD, Carboxylic acids and derivatives; BSD, Benzene and substituted derivatives; OOC, Organooxygen compounds; SSD, Steroids and steroid derivatives; FAs, Fatty acyls; Indoles, Indoles and derivatives; GPLs, Glycerophospholipids; **(C)** KEGG enrichment analysis of differential metabolites between NE and CK treatments. **(D)** Heatmap of differential metabolites associated with KEGG pathways (NE, healthy fruits with nitrogen excess; CK, healthy fruits with suitable nitrogen).

The Volcano plot (**Figure 2A**) showed that nitrogen excess led to differences in the fruit metabolites. After screening of differential metabolites (OPLS-DA VIP > 1 and P value < 0.1) detected 148 differential metabolites were found to be upregulated under nitrogen excess, which were grouped into seven main classes. These seven classed including carboxylic acids and derivatives (CAD), benzene and substituted derivatives (BSD), organooxygen compounds (OOC), steroids and steroid derivatives (SSD), fatty acyls (FAs), indoles and derivatives (Indoles), glycerophospholipids (GPLs). The CAD accounted for the highest number of differential metabolites (**Figure 2B**). The KEGG enrichment analysis showed that 5 pathways were enriched under nitrogen excess (**Figure 2C**): biosynthesis of amino acids, alanine, aspartate and glutamate metabolism, phenylalanine metabolism, arginine biosynthesis and aminoacyl-tRNA biosynthesis pathways. GABA, citrulline, arginine, phenylalanine and asparagine were significantly upregulated under nitrogen excess (**Figure 2D**). Indoleacetic acid, which is associated with growth, was significantly upregulated under nitrogen excess.

### 3.2 Effects of pest injury on fruit metabolism under different nitrogen levels

**Figure 3:**
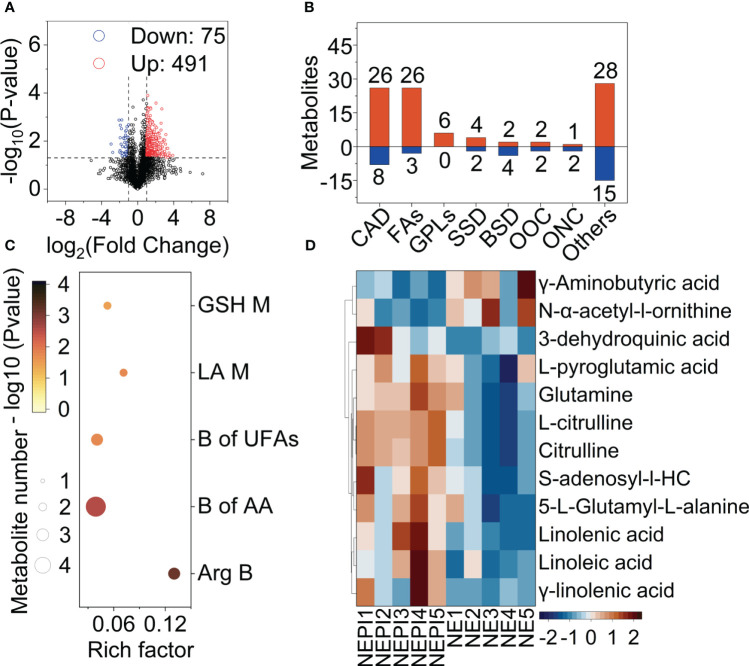
Overview of fruit metabolism changes in healthy (NE) and infested tomatoes (NEPI) under nitrogen excess. **(A)** Volcano map; the horizontal coordinate indicates the change in the metabolites (log_2_ fold change), and the vertical coordinate indicates the significance level (-log_10_ (P value)). The upregulated metabolites and downregulated metabolites are presented by red and blue dots, respectively. **(B)** Grouping classification of the upregulated and downregulated different metabolites. CAD: Carboxylic acids and derivatives. FAs, Fatty acyls; GPLs, Glycerophospholipids; SSD, Steroids and steroid derivatives; BSD, Benzene and substituted derivatives; OOC, Organooxygen compounds; ONC, Organonitrogen compounds. The numerals indicate the number of metabolites. **(C)** KEGG enrichment analysis of differential metabolites between NEPI and NE treatments. **(D)** Heatmap of differential metabolites associated with KEGG pathways (NEPI, infested fruits with nitrogen excess; NE, healthy fruits with nitrogen excess).

In total, 11782 metabolites were detected by LC-MS analysis, of which 491 metabolites and 75 metabolites were upregulated and downregulated by *B. dorsalis* feeding under nitrogen excess (**Figure 3A**). Screening of differential metabolites (OPLS-DA VIP > 1 and P value < 0.1) detected 131 pest injury (PI)-affected differential metabolites (**Figure 3B**). These differential metabolites were grouped into seven main classes: carboxylic acids and derivatives (CAD), fatty acyls (FAs), glycerophospholipids (GPLs), steroids and steroid derivatives (SSD), benzene and substituted derivatives (BSD), organooxygen compounds (OOC) and organonitrogen compounds (ONC).

The results of KEGG enrichment analysis in NEPI compared with NE showed 5 different enrichment pathways: glutathione metabolism, linoleic acid metabolism, biosynthesis of unsaturated fatty acids, biosynthesis of amino acids, and arginine biosynthesis (**Figure 3C**). A heatmap was drawn showing the contents and fold changes of 12 differential metabolites associated with the 5 pathways. L-Pyroglutamic acid, glutamine, citrulline, S-adenosyl-l-homocysteine, 5-L-glutamyl-L-alanine, linolenic acid, and linoleic acid were significantly upregulated in the NEPI group. In contrast to the above results, GABA was not significantly upregulated by NEPI treatment, but glutamine, which is a downstream substance of glutamate metabolism, was significantly upregulated by NEPI treatment (**Figure 3D**).

**Figure 4:**
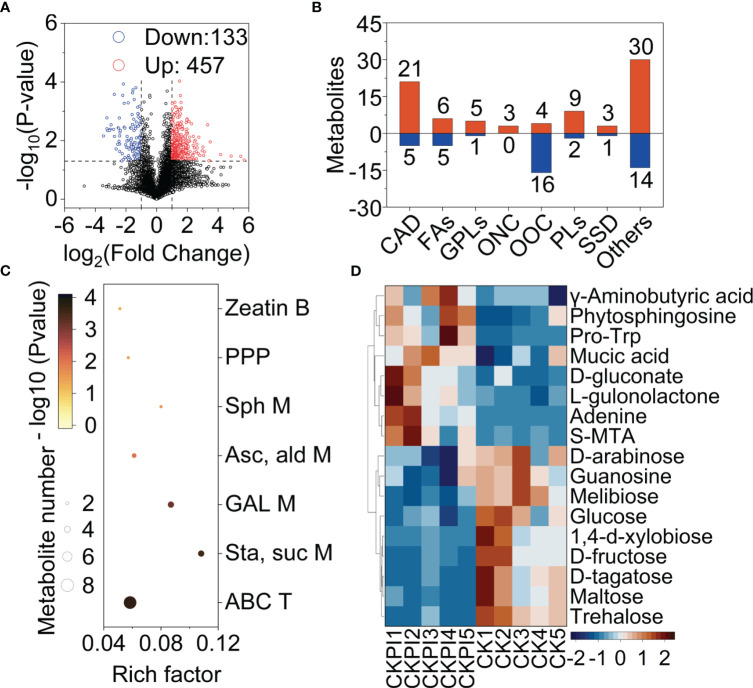
Overview of fruit metabolism changes in healthy (CK) and infested (CKPI) tomatoes of the suitable nitrogen group. **(A)** Volcano map; the horizontal coordinate indicates the change in the metabolites (log_2_ fold change), and the vertical coordinate indicates the significance level (-log_10_ (P value)). The upregulated metabolites and downregulated metabolites are presented by red and blue dots, respectively. **(B)** Grouping classification of the upregulated and downregulated differential metabolites. CAD, Carboxylic acids and derivatives; FAs, Fatty Acyls; GPLs, Glycerophospholipids; ONC, Organonitrogen compounds; OOC, Organooxygen compounds; PLs, Prenol lipids; SSD, Steroids and steroid derivatives. **(C)** KEGG enrichment analysis of differential metabolites between CKPI and CK treatments. **(D)** Heatmap analysis of differential metabolites associated with KEGG pathways (CKPI: infested fruits of suitable nitrogen, CK: healthy fruits of suitable nitrogen).

The volcano plot showed that 457 metabolites and 133 metabolites were upregulated and downregulated by injury from *B. dorsalis* in the CK group (**Figure 4A**). A total of 125 differential metabolites were grouped into seven main classes: carboxylic acids and derivatives (CAD), fatty acyls (FAs), glycerophospholipids (GPLs), organonitrogen compounds (ONC), organooxygen compounds (OOC), prenol lipids (PLs), steroids and steroid derivatives (SSD) (**Figure 4B**). The carboxylic acids and derivatives group still had the most differential metabolites. The KEGG analysis showed that differential metabolites were significantly enriched in 7 different enrichment pathways: zeatin biosynthesis, pentose phosphate pathway, sphingolipid metabolism, ascorbate and aldarate metabolism, galactose metabolism, starch and sucrose metabolism and ABC transporters (**Figure 4C**). The metabolic pathways were mainly associated with carbon metabolism. Arabinose, glucose, fructose and tagatose production were downregulated in the CKPI treatment. The content of GABA was significantly increased in the CKPI treatment (**Figure 4D**).

### 3.3 GABA is not a differential metabolite in the N deficiency group

**Figure 5:**
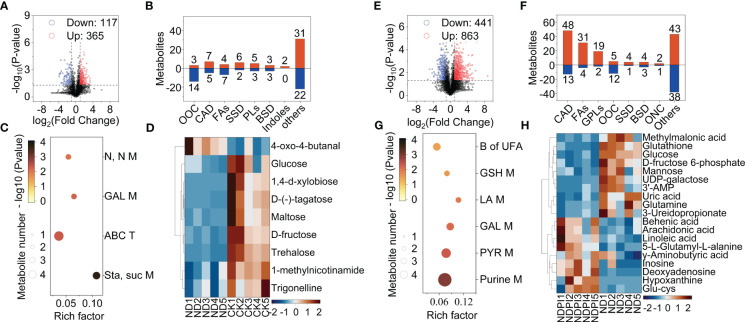
Overview of fruit metabolism changes under nitrogen deficiency (ND) and CK tomatoes. **(A)** Volcano map: the horizontal coordinate indicates the change in the metabolites (log_2_ fold change), and the vertical coordinate indicates the significance level (-log_10_ (P value)). The upregulated metabolites and downregulated metabolites are presented by red and blue dots, respectively. **(B)** Grouping classification of the upregulated and downregulated differential metabolites. OOC: Organooxygen compounds. CAD: Carboxylic acids and derivatives. FAs: Fatty acyls. SSD: Steroids and steroid derivatives. PLs: Prenol lipids. BSD: Benzene and substituted derivatives. Indoles: Indoles and derivative. **(C)** KEGG enrichment analysis of differential metabolites between ND and CK treatments. **(D)** Heatmap of differential metabolites associated with KEGG pathways (ND: healthy fruits with nitrogen deficiency, CK: healthy fruits with suitable nitrogen). Overview of fruit metabolism changes in healthy (ND) and infested tomatoes (NDPI) of nitrogen deficiency. **(E)** Volcano map; the horizontal coordinate indicates the change in the metabolites (log_2_ fold change), and the vertical coordinate indicates the significance level (-log_10_ (P value)). The upregulated metabolites and downregulated metabolites are presented by red and blue dots, respectively. **(F)** Grouping classification of the upregulated and downregulated differential metabolites. CAD, Carboxylic acids and derivatives; FAs, Fatty acyls; Gly, Glycerophospholipids; OOC, Organooxygen compounds; SSD, Steroids and steroid derivatives; BSD, Benzene and substituted derivatives; ONC, Organonitrogen compounds. **(G)** KEGG enrichment analysis of differential metabolites between NDPI and ND treatments. **(H)** Heatmap of differential metabolites associated with KEGG pathways ((NDPI, infested fruits with nitrogen deficiency; ND, healthy fruits with nitrogen deficiency).

The Volcano plot (**Figure 5A**) showed that nitrogen deficiency led to differences in the fruit metabolites. After screening of differential metabolites (OPLS-DA VIP > 1 and P value < 0.1) detected 117 differential metabolites were found to be upregulated under nitrogen deficiency, which were grouped into seven main classes. The organooxygen compounds (OOC) accounted for the highest number of differential metabolites (**Figure 5B**). The KEGG enrichment analysis showed that 4 pathways were enriched under nitrogen deficiency (**Figure 5C**): starch and sucrose metabolism, galactose metabolism, ABC transporters and nicotinate and nicotinamide metabolism. GABA was not a differential metabolite in nitrogen deficiency (**Figure 5D**).

The volcano plot showed that 863 metabolites and 441 metabolites were upregulated and downregulated by injury from *B. dorsalis* in the nitrogen deficiency group (**Figure 5E**). The 196 differential metabolites were divided into 8 classes: carboxylic acids and derivatives (CAD), fatty acyls (FAs), glycerophospholipids (GPLs), organooxygen compounds (OOC), steroids and steroid derivatives (SSD), benzene and substituted derivatives (BSD) and organonitrogen compounds (ONC) (**Figure 5F**). The KEGG analysis showed that most differential metabolites were significantly enriched in the biosynthesis of unsaturated fatty acids, glutathione metabolism, linoleic acid metabolism, galactose metabolism, pyrimidine metabolism and purine metabolism (**Figure 5G**). A heatmap was drawn showing the contents and fold changes of 18 differential metabolites associated with the 6 pathways. Deoxyadenosine and inosine, which are associated with energy metabolism, were upregulated in the NDPI treatment (**Figure 5H**). D-Fructose 6-phosphate, glucose and mannose, which are associated with galactose metabolism, were downregulated in the NDPI treatment. However, GABA was not significantly upregulated in the NDPI treatment, and it was not a differential metabolite in the nitrogen deficiency group.

### 3.4 Function of GABA in the interaction between nitrogen and *B. dorsalis* injury

**Figure 6:**
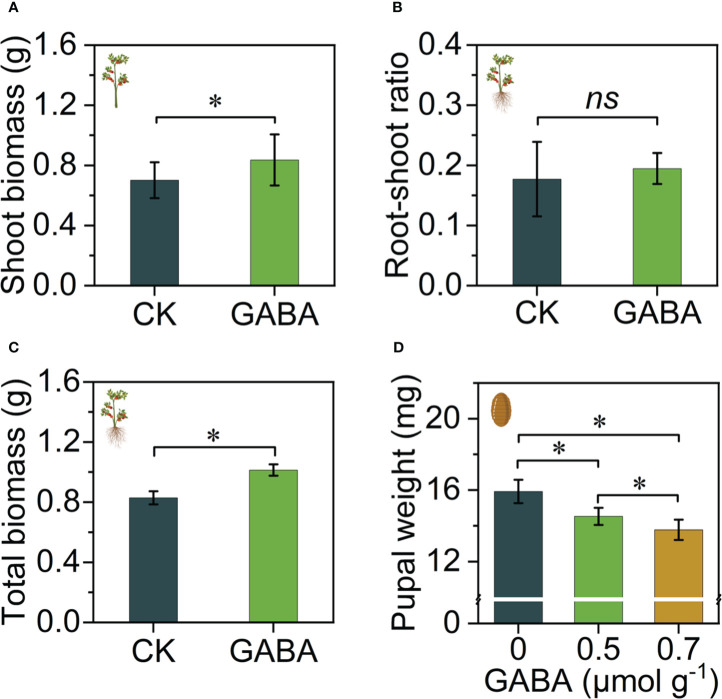
Effects of GABA supplements on the phenotypes of tomato biomass and the pupal biomass of (*B*) *dorsalis*. **(A)** The biomass of pre tomato shoots. **(B)** The root/shoot ratio of pre tomato. **(C)** The total biomass of pre tomato. **(D)** The pupal weight of *B. dorsalis*. The asterisk indicates significant differences (P < 0.05). The "ns" indicates no significant differences.

**Figure 7:**
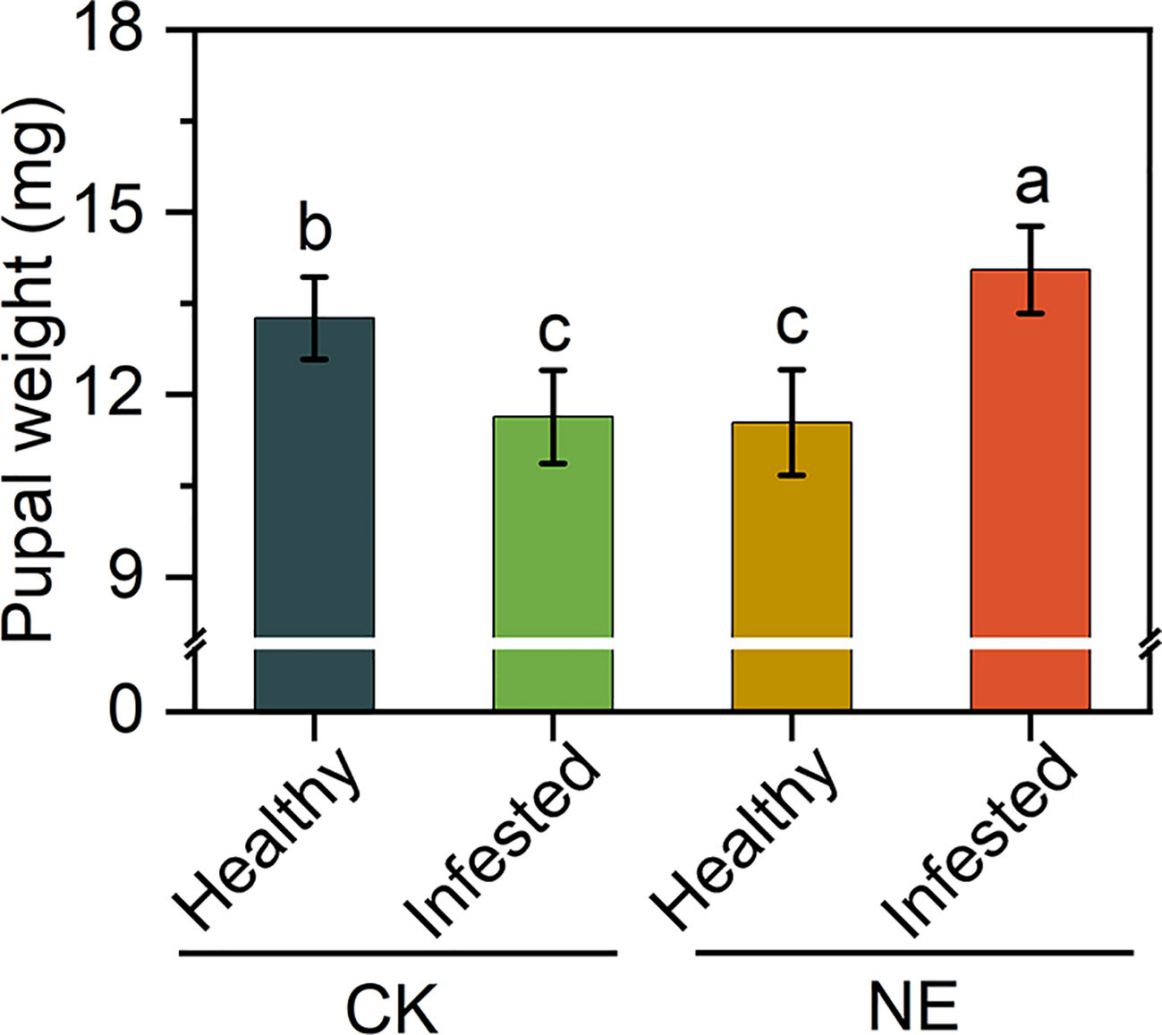
Effects of nitrogen excess on phenotypes of pupal biomass of *B. dorsalis* in healthy and infested fruits. The different letters in figure indicate significant differences between different treatments (P < 0.05).

The exogenous application of GABA significantly increased the shoot biomass and total biomass of tomato (**Figures 6A, C**), with no effect on the root/shoot ratio (**Figure 6B**). The feeding experiment showed that GABA significantly inhibited the pupal weight of *B. dorsalis*, and the inhibitory effect of 0.7 µmol/g GABA treatment was significantly higher than that of 0.5 µmol/g GABA treatment (**Figure 6D**). In healthy fruits without injury from *B. dorsalis*, the pupal weight (eggs of artificial inoculation) of the control was significantly higher than that of the nitrogen treatment. However, after injury of *B. dorsalis* (infested fruits), the pupal weight of the nitrogen excess treatment was significantly higher than that of the CK group (**Figure 7**).

## 4 Discussion

Some studies have reported that a high nitrogen supply increases the free amino acids in plants(Zhou et al., 2022). Plants use GABA, asparagine and arginine as nitrogen storage and transport amino acids (Kan et al., 2015). In particular, asparagine and arginine have higher nitrogen-to-carbon ratios and release and remobilize nitrogen after being hydrolyzed when the plant needs to mobilize nitrogen from source tissues (Hildebrandt et al., 2015). This study found that excess nitrogen promoted the biomass accumulation of tomato and *B. dorsalis*. The reason why high nitrogen increased the aboveground biomass of tomato was explained by the metabolomic results. First, it promoted primary metabolism (Atilio and Causin, 1996) and the accumulation of amino acids to promote the production of nutrients. We also found that key metabolites were significantly upregulated in the nitrogen excess treatment, particularly GABA and IAA. GABA connects the carbon and nitrogen metabolic fluxes in plants through the GABA shunt (Fait et al., 2008). Several studies have shown that GABA can promote pollen tube elongation and stem growth at low concentrations (Bouché and Fromm, 2004). In addition, IAA increases the accumulation of plant biomass. Ammonia nutrition can induce the activity of aldehyde oxidase and promote the synthesis of IAA (Koshiba et al., 1996).

As an inhibitory neuromuscular transmitter, GABA affects the normal development of insects by directly acting on GABA-gated chloride channels (Bown et al., 2006). The negative effects of GABA on insects have been verified in several studies. One study reared larvae with artificial diets containing GABA, and the feeding results showed that GABA significantly inhibited the growth of olive-banded leafler (*Choristoneura rosaceana*) larvae (Ramputh and Bown, 1996), and in another it significantly reduced the larval weight of *Spodoptera littoralis* (Scholz et al., 2015). The results of our research also showed that GABA had an inhibitory effect on the pupal weight of the fruit fly, and with increasing GABA concentration, the inhibition of biomass was more significant. Given the result of the upregulation of GABA content in nitrogen-excess fruits, ingestion of plant material that contains elevated GABA levels, could have negative consequences for insects (Bown et al., 2006). The growth of fruit fly larvae growing in nitrogen-excess fruits would in that case be inhibited, but the results were the opposite. Therefore, we also measured the metabolic changes of fruits that were cultured under different nitrogen levels with *B. dorsalis* injury to try to explain the reason for the change in biomass.

Experimental evidence supports the synthesis of GABA induced by herbivorous harm and involvement in plant defense (Bown et al., 2002; Mithofer and Boland, 2012). In plants, GABA biosynthesis is catalyzed by glutamate decarboxylase (GAD) (Akihiro et al., 2008). Plants initiate the signal transduction pathway when under environmental and biological stress, in which increased cytosolic Ca^2+^ stimulates Ca^2+^/calmodulin-dependent GAD activity and GABA synthesis (Zik et al., 2006). Research has demonstrated that GABA accumulation is stimulated by *Spodoptera littoralis* herbivory (Scholz et al., 2015). The crawling mechanical injury of *Choristoneura rosaceana* and *Heliothis virescens* can also induce the accumulation of GABA in a short time (Bown et al., 2002). GABA and glutamine are both downstream metabolites of glutamate metabolism. Glutamine synthetase (GS) catalyzes the synthesis of glutamine from free ammonium and glutamate. Some studies have shown that high-nitrogen nutrition can promote the activity of GS (Balotf and Kavoosi, 2016), and the assimilation of ammonium into glutamine is also one of the metabolic pathways which plants use to avoid the toxicity of high ammonium (Masclaux-Daubresse et al., 2006). Nitrogen-excess treatment provides tomato plants with a high concentration of ammonium, which is potentially toxic to plant cells. Free toxic ammonium in plants should be assimilated quickly to avoid ammonium toxicity (Linka and Weber, 2005). The main mechanism of ammonium assimilation is the GS/glutamine oxoglutarate aminotransferase (GOGAT) cycle. Ammonium can be assimilated into glutamine and glutamate via this cycle, which is the major pathway for primary nitrogen assimilation in plants (Lea and Miflin, 1974).

The metabolism of glutamate was the same in different treatment groups. The major reason is that plants seem to balance nitrogen metabolism by maintaining the homeostasis of glutamate. The concentration of glutamate in plants always remains relatively constant under different nitrogen levels in different culture conditions or in the diurnal cycle (Forde and Lea, 2007). High nitrogen input causes fluctuations in amino acids, including GABA, glutamine, arginine, asparagine, etc., in addition to glutamate. These metabolic changes are related to enzymes of nitrogen metabolism. Masclaux-Daubresse et al. (2006) argued that GS and glutamate dehydrogenase (GDH) were the major enzymes responsible for maintaining a constant concentration of glutamate (Masclaux-Daubresse et al., 2006), but it is also likely that the supply of 2-oxoglutarate is a key regulator (Forde and Lea, 2007). These studies explained that under high-nitrogen conditions, the content of glutamate in tomato fruit did not fluctuate, but the metabolism of GABA and glutamine fluctuated differently.

Glutamine is an important amino acid with the function of storing nitrogen, and an ammonium ion is fixed in the metabolic process of producing glutamine. *B. dorsalis* herbivory increases the levels of glutamate acid, phenylalanine and aspartic acid in tomato fruits with suitable nitrogen (Li et al., 2023). Our metabolomic results showed that injury to *B. dorsalis* changed the metabolic direction of glutamate and induced the upregulation of glutamine in the NE treatment group. The change in metabolic direction weakens the defense of tomato against *B. dorsalis* feeding. These results explain why the pupal weight of the fruit fly was increased in nitrogen-excess fruit that was injured by *B. dorsalis*. We believe that under the high nitrogen level, the change in glutamate metabolic direction was mediated by *B. dorsalis*, which may be the result of the joint action of carbon metabolism and nitrogen metabolism. Some reports have indicated that ammonium assimilation is closely related to carbon metabolism (Gauthier et al., 2010). The injury by *B. dorsalis* affected the carbon metabolism of plants, and high nitrogen affected their nitrogen metabolism, which destroyed the balance between carbon and nitrogen metabolism. Carbon metabolism provides the necessary energy and carbon framework for nitrogen metabolism. α-Ketoglutarate, the product of glucose metabolism and the TCA cycle, is an important carbon material in ammonium assimilation (Xun et al., 2020). GDH and GOGAT both catalyze the synthesis of glutamate from α-ketoglutarate, introducing a carbon framework into nitrogen metabolism (Masclaux-Daubresse et al., 2006). Some studies have shown that a sufficient carbon source allows plants to complete normal nitrogen metabolism and assimilate excessive ammonium (Vega-Mas et al., 2017). The damage by *B. dorsalis* significantly reduced the carbon source required for nitrogen metabolism. When the supply of the carbon framework is insufficient, plants need to seek efficient nitrogen assimilation pathways. Glutamine has a high nitrogen-to-carbon ratio (Kan et al., 2015); under this condition, ammonium assimilation is completed by glutamine synthetase. However, in the nitrogen-excess group, the KEGG pathway analysis did not show enrichment of the carbon metabolism pathway, possibly because the feedback of high nitrogen affects carbon metabolism. The results under nitrogen deficiency showed that the major differential metabolites were concentrated in carbon metabolism, and GABA was not a differential metabolite. Low nitrogen levels will significantly affect the carbon metabolism and nitrogen metabolism of plants, including the reduction of nitrogen-rich amino acid content (Zhou et al., 2021). Therefore, *B. dorsalis* did not induce significant metabolic changes in nitrogen-rich amino acids after harming nitrogen-deficient fruit. These results show that the GABA metabolome of plants defending against herbivorous harm depends on the level of nitrogen, making it a nitrogen-dependent defense response.

## 5 Conclusion

**Figure 8:**
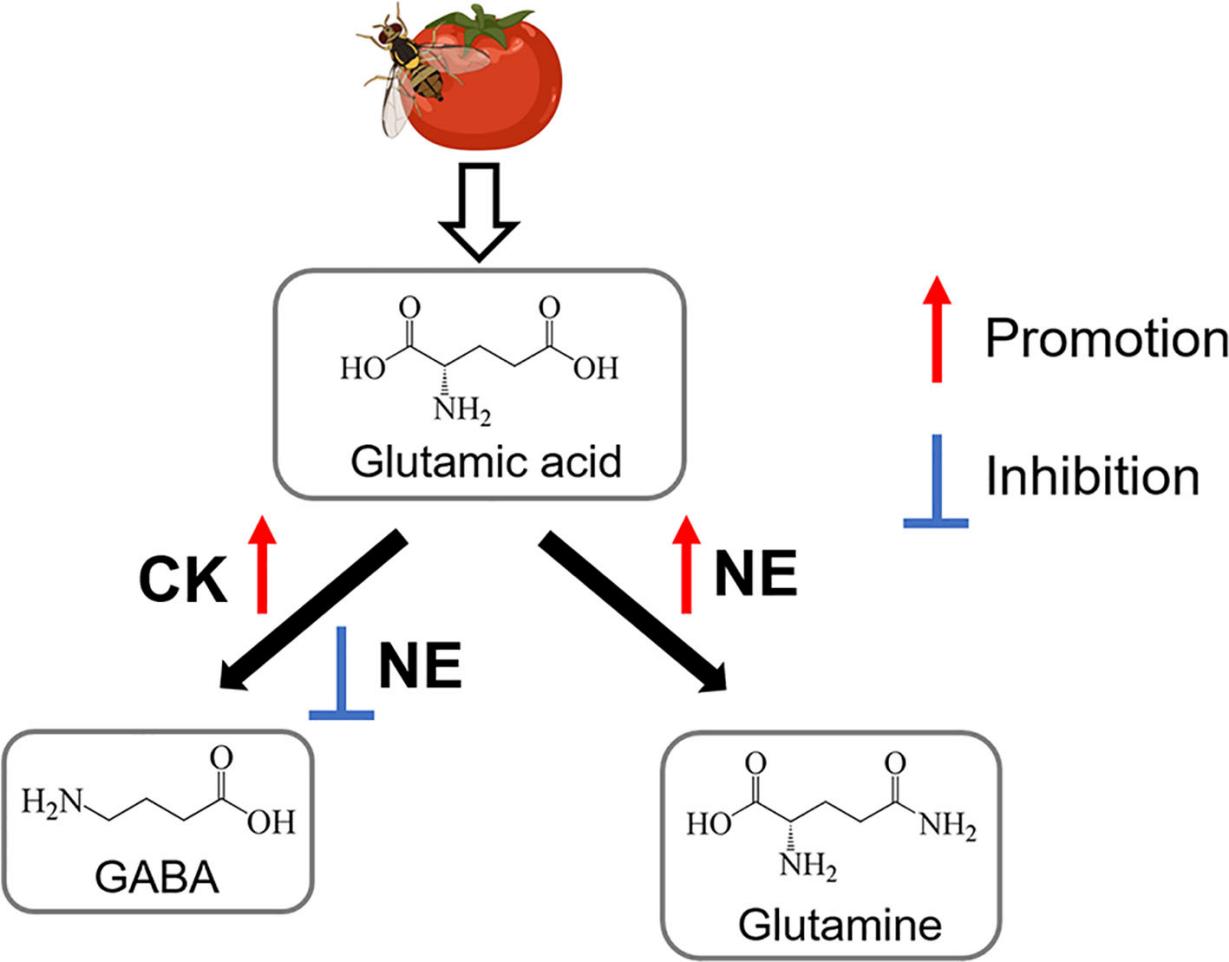
Model for metabolomic responses to nitrogen excess and *B. dorsalis* herbivory in tomato (CK, Suitable nitrogen; NE, nitrogen excess). Promotion and inhibition are presented by red and blue lines.

In conclusion, our research results supplement the research on the effect of high nitrogen on plant defense. Nitrogen excess promoted the aboveground biomass accumulation of tomato and *B. dorsalis*. According to our metabolomic results, fruits that are cultured in suitable nitrogen levels can promote the synthesis of GABA under biological stress. We also demonstrated the control effect of GABA on *B. dorsalis*. GABA supplementation not only increased the aboveground biomass of plants but also improved the defensive response of tomato. Injury by *B. dorsalis* inhibited the biosynthesis of GABA, but injury promoted the biosynthesis of glutamine in nitrogen-excess fruits (**Figure 8**). The study showed that excess nitrogen inhibited the defense response of tomato to biological stress. These findings provide a foundation for future research on the nitrogen-mediated interaction between insects and plants.

## Data availability statement

The original contributions presented in the study are included in the article/**Supplementary Material.** Further inquiries can be directed to the corresponding author.

## Ethics statement

Ethical review and approval was not required for the study on animals in accordance with the local legislation and institutional requirements.

## Author contributions

HaL, YZ, and ZZ contributed to conception and design of the study. HaL organized the database. HaL and YZ performed the statistical analysis. HaL wrote the first draft of the manuscript. YZ wrote sections of the manuscript. All authors contributed to the article and approved the submitted version.
