# Supplementary material for: The nitrogen-dependent GABA pathway of tomato provides resistance to a globally invasive fruit fly
Source: Front Plant Sci. 2023 Dec 7;14:1252455. doi: 10.3389/fpls.2023.1252455 (PMC10751092; doi:10.3389/fpls.2023.1252455)
Supplement: Supplementary file 1 [file DataSheet_1.docx]

***Frontiers in Plant Science***

**Supporting Information**

**The nitrogen-dependent GABA pathway of tomato provides resistance to a globally invasive fruit fly**

**Hao Li^1,2*^, Yuan Zhang^1,2*^,** **Hu Li^1^, Gadi VP Reddy^3^, Zhihong Li^1,2^,** **Fajun Chen^4^, Yucheng Sun^5^, Zihua Zhao^1, 2, 🖂^**

**🖂Correspondence**

Zihua Zhao, Department of Plant Biosecurity, College of Plant Protection, China Agricultural University, No.2 Yuanmingyuan West Road, Beijing, 100193, China.

Email: [zhzhao@cau.edu.cn](mailto:zhzhao@cau.edu.cn)

Tel: +86 010-62732068

**LC–MS analysis methods**

For HILIC separation, samples were analyzed using a 2.1 mm × 100 mm ACQUIY UPLC BEH 1.7 µm column (waters, Ireland). In both ESI positive and negative modes, the mobile phase contained A=25 mM ammonium acetate and 25 mM ammonium hydroxide in water and B= acetonitrile. The gradient was 85% B for 1 min and was linearly reduced to 65% in 11 min, and then was reduced to 40% in 0.1 min and kept for 4 min, and then increased to 85% in 0.1 min, with a 5 min reequilibration period employed. The ESI source conditions were set as follows: Ion Source Gas1 (Gas1) as 60, Ion Source Gas2 (Gas2) as 60, curtain gas (CUR) as 30, source temperature: 600℃, IonSpray Voltage Floating (ISVF)±5500 V. In MS only acquisition, the instrument was set to acquire over the m/z range 60-1000 Da, and the accumulation time for TOF MS scan was set at 0.20 s/spectra. In auto MS/MS acquisition, the instrument was set to acquire over the m/z range 25-1000 Da, and the accumulation time for product ion scan was set at 0.05 s/spectra. The product ion scan is acquired using information dependent acquisition (IDA) with high sensitivity mode selected. The parameters were set as follows: the collision energy (CE) was fixed at 35 V with ± 15 eV; declustering potential (DP), 60 V (+) and −60 V (−); exclude isotopes within 4 Da, candidate ions to monitor per cycle: 10 (From Shanghai Applied Protein Technology Co., Ltd.)
